# Efficacy and Safety of a Brain-Penetrant Biologic TNF-α Inhibitor in Aged APP/PS1 Mice

**DOI:** 10.3390/pharmaceutics14102200

**Published:** 2022-10-16

**Authors:** Weijun Ou, Yuu Ohno, Joshua Yang, Devaraj V. Chandrashekar, Tamara Abdullah, Jiahong Sun, Riley Murphy, Chuli Roules, Nataraj Jagadeesan, David H. Cribbs, Rachita K. Sumbria

**Affiliations:** 1 Department of Biomedical and Pharmaceutical Sciences, School of Pharmacy, Chapman University, Irvine, CA 92618, USA; 2 Henry E. Riggs School of Applied Life Sciences, Keck Graduate Institute, 535 Watson Dr, Claremont, CA 91711, USA; 3 Crean College of Health and Behavioral Sciences, Chapman University, Irvine, CA 92618, USA; 4 MIND Institute, University of California, Irvine, CA 92697, USA; 5 Department of Neurology, University of California, Irvine, CA 92868, USA

**Keywords:** Alzheimer’s disease, transferrin receptor, TNF-α inhibitor, blood–brain barrier, molecular Trojan horse

## Abstract

Tumor necrosis factor alpha (TNF-α) plays a vital role in Alzheimer’s disease (AD) pathology, and TNF-α inhibitors (TNFIs) modulate AD pathology. We fused the TNF-α receptor (TNFR), a biologic TNFI that sequesters TNF-α, to a transferrin receptor antibody (TfRMAb) to deliver the TNFI into the brain across the blood–brain barrier (BBB). TfRMAb-TNFR was protective in 6-month-old transgenic APP/PS1 mice in our previous work. However, the effects and safety following delayed chronic TfRMAb-TNFR treatment are unknown. Herein, we initiated the treatment when the male APP/PS1 mice were 10.7 months old (delayed treatment). Mice were injected intraperitoneally with saline, TfRMAb-TNFR, etanercept (non-BBB-penetrating TNFI), or TfRMAb for ten weeks. Biologic TNFIs did not alter hematology indices or tissue iron homeostasis; however, TfRMAb altered hematology indices, increased splenic iron transporter expression, and increased spleen and liver iron. TfRMAb-TNFR and etanercept reduced brain insoluble-amyloid beta (Aβ) 1-42, soluble-oligomeric Aβ, and microgliosis; however, only TfRMAb-TNFR reduced Aβ peptides, Thioflavin-S-positive Aβ plaques, and insoluble-oligomeric Aβ and increased plaque-associated phagocytic microglia. Accordingly, TfRMAb-TNFR improved spatial reference memory and increased BBB-tight junction protein expression, whereas etanercept did not. Overall, despite delayed treatment, TfRMAb-TNFR resulted in a better therapeutic response than etanercept without any TfRMAb-related hematology- or iron-dysregulation in aged APP/PS1 mice.

## 1. Introduction

Alzheimer’s disease (AD) is a chronic and progressive neurodegenerative disease characterized by extracellular amyloid-beta (Aβ) plaques and intraneuronal neurofibrillary tangles [1]. Besides Aβ and tau tangles, mounting evidence suggests a central role of neuroinflammation in AD pathogenesis [2]. Neuroinflammation is an inflammatory response to a pathological stimulus in the brain orchestrated by the cerebral immune cells, primarily the microglia [2]. In the AD brain, Aβ aggregates trigger microglial activation, which results in the release of pro-inflammatory cytokines, including tumor necrosis factor alpha (TNF-α) [2,3]. Accordingly, elevated serum levels of TNF-α in AD patients correlate with increased physical and cognitive impairment [4,5], and TNF-α is widely studied as a target for AD treatment [6]. Further, a genome-wide association study demonstrated that TNF-α polymorphisms that are associated with inflammatory diseases and elevated TNF-α levels are linked with late-onset AD [7].

Several biologic TNF-α inhibitors have been approved for autoimmune conditions and have been tested on AD rodent models and in humans. To achieve TNF-α inhibition within the AD brain, biologic TNF-α inhibitors are administered via different routes or at high doses to bypass the blood–brain barrier (BBB) due to their limited BBB penetration [6]. For example, infliximab, a bivalent IgG monoclonal antibody acting as a competitive inhibitor to TNF-α, reduced Aβ and tau pathology [8] and improved visual recognition memory [9] upon intracerebroventricular injection in transgenic mouse models of AD. A woman AD patient experienced rapid cognitive improvement upon intrathecal administration of infliximab [10]. Similarly, etanercept, a fusion protein consisting of the fragment crystallizable (Fc) region of human IgG1 and the extracellular ligand-binding domain of the type-II human TNF-α receptor (TNFRII), improved clinical measures in AD patients when given by the perispinal route [11]. High-dose (30 mg/kg) peripheral (subcutaneous) etanercept administration reduced Aβ-associated pathology in a non-transgenic mouse model of Aβ-induced cognitive deficit [12]. However, a double-blinded phase 2 trial showed no clinical benefit of peripheral (subcutaneous) etanercept administration in AD patients [13].

To bypass the BBB and enter the brain parenchyma, the biologic TNF-α inhibitor of choice can be fused to a monoclonal antibody against the mouse transferrin receptor (TfRMAb); the latter undergoes receptor-mediated transcytosis from the blood into the brain across the BBB [14]. A fusion protein of TfRMAb and the extracellular domain of human TNFRII (TNFR), a TNF-α inhibitor, was engineered [14]. The TfRMAb-TNFR fusion protein enters the mouse brain following intravenous, subcutaneous, and intraperitoneal administration owing to the TfRMAb domain, and the TNFR domain of the fusion protein binds to TNF-α to block downstream TNF-α signaling [15]. The TfRMAb-TNFR fusion protein enters the brain with an uptake of ~3% injected dose/gram brain post intravenous injection; in contrast, the brain uptake of the OX26 monoclonal antibody that does not recognize the mouse TfR is negligible in mice [16]. TfRMAb-TNFR is, therefore, a brain-penetrant biologic TNF-α inhibitor that rapidly enters the brain via the transvascular route without the need for invasive administration or high doses [14,15].

Our previous work with the TfRMAb-TNFR in six-month-old mutant APP/PS1 male mice showed better therapeutic indices for the TfRMAb-TNFR fusion protein in comparison to the non-BBB penetrating etanercept [17]. There was a significant reduction in brain Aβ burden, intercellular adhesion molecule-1 (neuroinflammatory marker), brain parenchymal IgG (BBB damage marker), and recognition memory deficits in the adult APP/PS1 mice treated with the TfRMAb-TNFR [17]. Further, TfRMAb-TNFR fusion protein treatment resulted in low antidrug-antibody formation, which was comparable to etanercept after twelve weeks of chronic dosing, with no signs of an immune response or cerebral microhemorrhage development [17]. Furthermore, the antidrug-antibodies formed against TfRMAb-TNFR are expected to be non-neutralizing as no changes in TfRMAb-TNFR plasma concentrations or clearance were observed following chronic dosing [18]. However, the effect of initiating TfRMAb-TNFR treatment in older APP/PS1 mice when the AD pathology is full-blown, and its safety profile following chronic administration in comparison to TfRMAb alone, have not been studied and were the focus of the current investigation. The former is important given that AD is a progressive neurodegenerative disease that advances with age [1], and the latter is crucial due to the reported hematology- and iron-related dysregulation with chronic TfRMAb dosing [19] and because TfRMAb-based fusion proteins have now advanced to humans [20]. Therefore, in the current study, we used older 10.7-month-old APP/PS1 male mice (instead of the 6-month-old male APP/PS1 mice used in our prior work [17]) to mimic a delayed-treatment regimen and evaluated the effect of the TfRMAb-TNFR on spatial reference memory, Aβ load, and microgliosis following ten weeks of treatment. Further, the effect of chronic TfRMAb-TNFR dosing on hematology indices, iron transporter expression, and tissue iron levels was studied in comparison with TfRMAb.

## 2. Materials and Methods

### 2.1. Fusion Protein

Recombinant TfRMAb-TNFR was produced via transient expression in Chinese hamster ovary (CHO)-K1 cells, sequentially purified by protein A and size exclusion chromatography (WuXi Biologics, Cranbury, NJ, USA), and verified by immunoblot as described previously [17,18]. The affinity of TfRMAb-TNFR to the mouse TfR and human TNF-α was confirmed by enzyme-linked immunoassays (ELISAs) [18]. Both the TfRMAb-TNFR fusion protein and etanercept (International Laboratory USA, San Francisco, CA, USA) were formulated in 100 mM glycine, 150 mM NaCl, 28 mM Tris, and 0.01% Polysorbate 80, pH = 6.4, sterile filtered, and stored at −80 °C until use. The chimeric TfRMAb (Genscript, Piscataway, NJ, USA) was produced via transient expression in ExpiCHO cells, sequentially purified by a protein G column and size exclusion chromatography, and verified by immunoblot, high performance liquid chromatography, and for endotoxin [19]. TfRMAb was formulated in 10 mM sodium acetate, 150 mM NaCl, and 0.01% polysorbate 80, pH = 6, sterile filtered, and stored at −80 °C until use, as previously described [19].

### 2.2. Chronic Dosing in a Mouse Model of Human Amyloidosis

Animal studies were performed on the 10.7-months-old hemizygous (APPswe, PSEN1dE9) APP/PS1 male mice (Jackson Laboratories, Bar Harbor, ME, USA) in compliance with University Laboratory Animal Resources under protocols approved by the University of California, Irvine, Institutional Animal Care, and Use Committee. All the mice were housed in standard cages under 12 h light/12 h dark cycles, with constant free access to food and water, and divided into the following four groups: APP/PS1-Saline (treated with saline; *n* = 20), APP/PS1-TfRMAb-TNFR (treated with 3 mg/kg TfRMAb-TNFR; *n* = 10), APP/PS1-Etanercept (treated with 1.5 mg/kg etanercept; *n* = 10) and APP/PS1-TfRMAb (treated with 2.25 mg/kg TfRMAb; *n* = 5). The doses were based on the amino acid sequence of TfRMAb-TNFR, which is 25% TNFR and 75% TfRMAb [14,16]. Etanercept is 50% TNFR based on its amino acid sequence [21]. Mice were injected intraperitoneally (IP) with the respective agents three days per week for ten weeks. All injected mice were carefully observed to check for any abnormal response to the treatment (general appearance, spontaneous locomotion, posture, and breathing) after each injection, and their body weights were monitored weekly [19]. After ten weeks of treatment, locomotion and spatial reference memory were assessed using the open-field and Y-maze tests, respectively. Terminal blood was collected at 11 weeks, and a subset of blood samples per group was shipped out on ice for a complete blood count (Molecular Diagnostic Services, Inc., San Diego, CA, USA), after which mice were euthanized with Euthasol (150 mg/kg, IP) and perfused with cold phosphate-buffered saline (PBS). After mice were sacrificed (the age of mice at sacrifice was 13 months), brains were excised, the right hemi-brains were immediately fixed in 4% paraformaldehyde for immunostaining, and the left hemi-brains were frozen in dry ice for ELISA and Western blotting.

### 2.3. Open-Field Testing

The open-field test was performed after ten weeks of treatment, as described previously, using a square-shaped white open-field box (72 cm × 72 cm with 36 cm walls) with a center square (36 cm × 36 cm) to measure locomotion and exploration [18]. Briefly, each mouse was gently placed in the white open-field box, and their movements were recorded for 5 min. Resting time, mean speed, and distance traveled were measured by the SMART Video Tracking Software (Panlab, Harvard Apparatus, Holliston, MA, USA) [18].

### 2.4. Modified Y-Maze

The modified Y-maze was used to evaluate the spatial reference memory in aged APP/PS1 mice [18]. The Y-maze apparatus consisting of three radial 30 cm arms (start arm, novel arm, and familiar arm), was placed at the ground level. During the training phase, mice were placed in the start arm to face the center of the maze and allowed to locate and explore only the start and familiar arms for 8 min, while a removable door blocked the novel arm. After a 30 min interval, the door blocking the novel arm was removed, and the mice were placed into the start arm again and allowed to explore the three arms for an additional 8 min during the testing phase. Mice that did not explore the familiar arm during the training phase or did not leave the start arm during the training or testing phase were excluded from the analysis. Time and distance in the novel arm, latency to the novel arm, and percentage of mice selecting the novel arm as the first arm choice were measured using the SMART Video Tracking Software (Panlab, Harvard Apparatus, Holliston, MA, USA).

### 2.5. Brain Tissue Cryosection

The right hemi-brain of each mouse was fixed with 4% paraformaldehyde in PBS for 24 h, then serially cryoprotected in 10%, 20%, and 30% sucrose solution at 4 °C for 24 h each, followed by freezing at −80 °C. The frozen hemi-brains were mounted using the Tissue-Tek optimal-cutting temperature compound (Fisher Scientific, Waltham, MA, USA) and cut into 20 μm sagittal sections using a freezing cryostat (Micron Instruments, Simi Valley, CA, USA). Three sections (600 µm apart) per mouse were used for immunostaining as described below.

### 2.6. Aβ Detection

Alexa Fluor 488-conjugated 6E10 monoclonal antibody (BioLegend, San Diego, CA, USA) and Thioflavin-S (Thio-S) (Sigma-Aldrich, St. Louis, MO, USA) staining were used to stain Aβ peptides and senile plaques, respectively, as described previously [17]. For Aβ immunofluorescence, free-floating brain sections were washed in PBS for 5 min and blocked with 0.5% bovine serum albumin (BSA) in PBS containing 0.3% TritonX-100 for 1 h at room temperature (RT), then stained for Aβ peptides with 1 μg/mL of Alexa Fluor 488-conjugated 6E10 monoclonal antibody overnight at 4 °C after epitope exposure using 70% formic acid for 10 min at RT. Thio-S staining was performed with 1% Thio-S solution in 80% ethanol for 15 min on mounted sections which had been sequentially washed with 70% and 80% ethanol for 1 min thrice. Slides were then sealed with the Vectamount aqueous mounting media (Vector Laboratories, Newark, CA, USA) and stored at 4 °C until imaging. The Thio-S- or 6E10-stained brain tissue sections were imaged using a BZ-X710 Keyence fluorescence microscope (Keyence, Itasca, IL, USA) under a 2 × objective to capture the entire brain tissue section in one image, and digitized images were analyzed using the NIH ImageJ software (version 1.53e), National Institutes of Health, Bethesda, MD, USA (https://imagej.nih.gov/ij) as published previously [22].

### 2.7. Microglial Immunostaining

Free-floating sagittal brain sections from each mouse were washed three times in PBS for 5 min, followed by antigen retrieval in 10 mM sodium citrate buffer (10 mM sodium citrate, 0.05% Tween 20, pH 6.0) at 90 °C for 15 min. Brain sections were washed twice in distilled water for 5 min and blocked with 0.5% BSA in PBS containing 0.3% Triton X-100 for 60 min at RT. The tissue sections were incubated with a primary antibody solution consisting of both 15 µg/mL anti-mouse Axl polyclonal goat antibody (R&D system, Minneapolis, MN, USA) and 0.5 µg/mL anti-ionized calcium binding adaptor molecule 1 (Iba1) polyclonal rabbit antibody (Wako, Richmond, VA, USA) in PBS containing 0.3% Triton X-100 and 0.5% BSA overnight at 4 °C. The tissue sections were washed three times in PBS for 5 min and incubated in the dark with a secondary antibody solution consisting of both 0.2% Alexa fluor 594 donkey anti-goat IgG (Thermo Fisher Scientific, Waltham, MA, USA) and 0.1% Alexa fluor 488 donkey anti-rabbit IgG (BioLegend; San Diego, CA, USA) in PBS containing 0.3% Triton X-100 and 0.5% BSA for 2 h at RT. The sections were then washed three times in PBS for 10 min and rinsed in distilled water. The sections were mounted onto glass slides, coverslipped with Vectamount aqueous mounting media (Vector Laboratories, Newark, CA, USA), and sealed with nail polish. Slides were stored at 4 °C until imaging.

### 2.8. Axl and Iba1 Quantification

Microgliosis was assessed using the Iba1 immunostaining, and phagocytic microglia were identified by immunostaining for Axl, a phagocytic receptor expressed on microglial cells [23,24]. For *Axl* and Iba1 double-label immunostaining, three distinct brain sections were selected for analysis for each mouse from the experimental groups except for the saline-treated APP/PS1 mice, from which a random subset of saline mice was used for the analysis. For each brain section, two regions in the cerebral cortex and one region in the hippocampus were imaged using a Nikon Ti-E Confocal Microscope (Nikon Instruments Inc., Melville, NY, USA) at a 40× magnification. Mature Aβ plaques were detected by the autofluorescence generated by the β-sheet-rich structures in Aβ plaques under ultra-violet (UV) light (405 nm) [25]. Iba1 was visualized using the blue laser (green), Axl was visualized using the green laser (red), and the Aβ plaques were visualized under UV light (blue). The digitized images were analyzed using NIH ImageJ (version 1.53e, Bethesda, MD, USA) using a threshold setting to calculate tissue area positive for Axl, Iba1, or Aβ plaques [18]. For microgliosis, total and plaque-associated microglia were quantified. To determine plaque-associated microgliosis, only microglia associated with plaques were outlined and included in the analysis. For this, immunofluorescent images were converted to the RGB format, and the threshold values of images were manually calibrated to eliminate background noise [18]. After adjusting the threshold, the “analyze” function was used to report the tissue area positive for Axl, Iba1, or Aβ plaques. To determine the microglial association with Aβ plaques, the plaque-associated Iba1-positive area was normalized to the plaque area and total Iba1 area to yield the plaque-associated microglia ratio. Similarly, to determine the phagocytic phenotype of the microglia associated with the Aβ plaques, the area of the Axl-positive microglia was normalized to the plaque area and the Iba1 area to yield the plaque-associated Axl ratio. Three investigators blinded to the experimental groups performed all the ImageJ analyses.

### 2.9. Quantification of Aβ (1-42) and Oligomeric Aβ by ELISA

The left hemi-brain cerebrum (without the cerebellum) was used for the Aβ (1-42) and high-molecular-weight oligomeric Aβ ELISA. Briefly, pulverized cerebrums were homogenized in 10 volumes of homogenization buffer (50 mM Tris-HCl, pH 7.6, 150 mM NaCl, 1 tablet/10 mL of Roche complete ethylenediaminetetraacetic acid (EDTA)-free Mini protease inhibitor, 5 mM EDTA, 2 mM 1,10-phenanthroline; tris-buffered saline (TBS) buffer) and centrifuged at 100,000× *g* for 1 h at 4 °C. The supernatant (TBS-soluble fraction) was used to measure soluble high-molecular-weight oligomeric Aβ (>30 kDa). The pellet was further homogenized in 10 volumes of homogenization buffer (5 M guanidine HCl (Gu-HCl), 0.05 M Tris, pH = 8.0) and rotated for 3 h at RT. The homogenate was centrifuged at 20,800× *g* for 15 min at RT, and the supernatant (Gu-HCl-soluble fraction) was used to measure insoluble Aβ (1-42) and insoluble high-molecular-weight oligomeric Aβ (>30 kDa) species. Protein assay was performed by bicinchoninic acid kit (Pierce Chemical Co., Rockford, IL, USA), and immunoreactive human Aβ (1-42) and oligomeric Aβ were measured using the Aβ (1-42) human ELISA Kit (Thermo Fisher Scientific, Waltham, MA, USA) and the high-molecular-weight oligomeric Aβ ELISA kit (Wako, Richmond, VA, USA), respectively.

### 2.10. Western Blot Analysis

Protein was extracted from the brain, spleen, and liver using the Radio-Immunoprecipitation Assay (RIPA) buffer or TBS with Pierce Protease Inhibitor (Thermo Fisher Scientific, Waltham, MA, USA) as previously described [19]. Briefly, a subset of the pulverized brains was homogenized in 10 volumes of RIPA (25 mM Tris-HCL, pH 7.6, 150 mM NaCl, 1% NP-40, 1% sodium deoxycholate, 0.1% SDS with 5 mM EDTA) or TBS and centrifuged at 20,800× *g* for 20 min at 4 °C. The supernatants were processed with 4× Laemmli buffer (Bio-Rad, Hercules, CA, USA) with 10% beta-mercaptoethanol by boiling for 10 min. Protein samples (30–50 μg) were separated on 4–20% sodium dodecyl sulfate (SDS) ready precast gels (Bio-Rad, Hercules, CA, USA) and transferred to Polyvinylidene fluoride (PVDF) membranes (Bio-Rad, Hercules, CA, USA). Membranes were probed at 4 °C overnight with anti-mouse zonula occludens-1 (ZO-1) rabbit polyclonal antibody (1:1000 in 3% milk, Thermo Fisher Scientific, Waltham, MA, USA), anti-human amyloid precursor protein (APP) mouse monoclonal antibody (1:1000 in 3% milk, BioLegend, San Diego, CA, USA), anti-claudin-5 mouse monoclonal antibody (1:1000 in 3% milk, Santa Cruz Biotechnology, Dallas, TX, USA), anti-β-site APP cleaving enzyme 1 (BACE1) rabbit polyclonal antibody (1:1000 in 3% milk, Thermo Fisher Scientific, Waltham, MA, USA), anti-IκBα mouse monoclonal antibody (1:1000 in 3% milk, Cell Signaling Technology, Danvers, MA, USA), anti-TfR-1 mouse monoclonal antibody (1:1000 in 3% milk, Thermo Fisher Scientific, Waltham, MA, USA), anti-TfR-2 mouse monoclonal antibody (1:1000 in 3% milk, Thermo Fisher Scientific, Waltham, MA, USA) and anti-ferroportin rabbit polyclonal antibody (1:1000 in 3% milk, Novus Biologicals, Centennial, CO, USA). Membranes were probed with anti-mouse IgG kappa, horseradish peroxidase (HRP)-linked antibody (Santa Cruz Biotechnology, Dallas, TX, USA) or anti-rabbit IgG, HRP-linked antibody (Cell Signaling Technology, Danvers, MA, USA) for 1 h at RT. Finally, membranes were probed with an anti–β-actin antibody (Santa Cruz Biotechnology, Dallas, TX, USA) as a loading control. Chemiluminescence was detected using the Azure C500 gel imager (Azure Biosystems, Dublin, CA, USA), and NIH ImageJ (version 1.53e, Bethesda, MD, USA) was used to quantify the intensity of Western blot bands. All the values were normalized to APP/PS1-Saline mice.

### 2.11. Tissue Iron Measurements

For all the mice, a part of the left hemi-brain, spleen, and liver was processed to determine tissue iron levels using the Agilent 8900 triple quadrupole Inductively Coupled Plasma Mass Spectrometer (ICP-MS; Agilent, Lomita, CA, USA), as described previously, at the Pomona College Environmental Analysis Laboratories [19]. Briefly, tissue samples were digested in 67% nitric acid overnight at RT, followed by digestion using 30% hydrogen peroxide for 1 h at 90 °C before running the samples using the ICP-MS.

### 2.12. Statistical Analysis

Data are represented as mean ± standard deviation (SD), and all statistical analysis was performed using GraphPad Prism 9 (GraphPad Software Inc., San Diego, CA, USA). Outliers were identified using the Grubb’s test, and the normality of numerical variables was assessed using the Kolmogorov–Smirnov test. One-way analysis of variance (ANOVA) with Holm Sidak’s post hoc test was used to compare normal numerical data. The % of mice selecting the novel arm (nominal data) was analyzed using the Fisher’s exact test, and the Pearson correlation was used for correlation analysis. A two-tailed *p* < 0.05 was considered statistically significant.

## 3. Results

### 3.1. Chronic TfRMAb-TNFR Dosing Reduced Aβ Load in Aged APP/PS1 Mice

**Figure 1:**
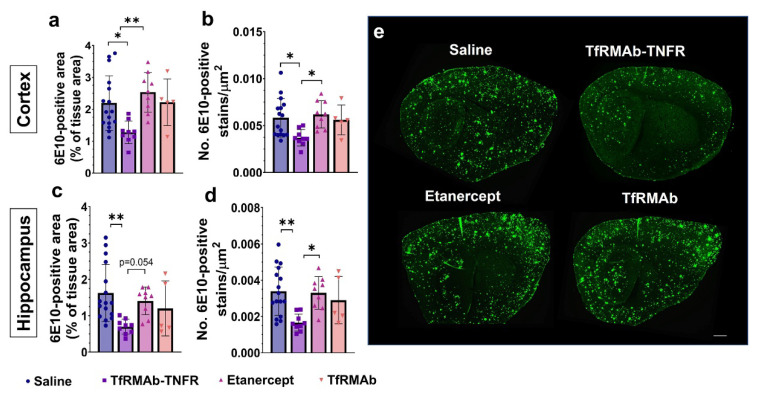
Effect of TfRMAb-TNFR dosing on 6E10-positive Aβ load. Cortical 6E10-positive Aβ area (**a**) and puncta number (**b**) were significantly lower in the TfRMAb-TNFR-treated mice compared to the saline-treated mice. There was a significant reduction in the hippocampal 6E10-positive Aβ area (**c**) and puncta number (**d**) in the TfRMAb-TNFR-treated mice compared to the saline-treated mice. Etanercept-treated mice had a higher 6E10-positive area and puncta number in the cortex and a higher puncta number in the hippocampus compared to the TfRMAb-TNFR-treated mice. 6E10-positive Aβ stains are shown in the representative sagittal brain section images in (**e**). Scale bar = 200 µm. Data are presented as mean ± SD of *n* = 5–16 per treatment group, and data were analyzed using the one-way ANOVA with the Holm Sidak’s post hoc test. * *p* < 0.05, ** *p* < 0.01 for the indicated comparisons.

Chronic TfRMAb-TNFR treatment significantly reduced cortical 6E10-positive Aβ area (*p* < 0.05; Figure 1a,e) and 6E10-positive puncta number (*p* < 0.05; Figure 1b,e) compared to the saline-treated mice. TfRMAb-TNFR treatment also significantly reduced hippocampal 6E10-positive Aβ area (*p* < 0.01; Figure 1c,e) and 6E10-positive puncta number (*p* < 0.01; Figure 1d,e) compared to the saline-treated mice. Etanercept or TfRMAb treatment did not reduce the 6E10-positive Aβ load in the aged APP/PS1 mice compared to the saline-treated mice. Etanercept-treated mice had significantly higher cortical 6E10-positive Aβ area (*p* < 0.01; Figure 1a,e) and 6E10-positive puncta number (*p* < 0.05; Figure 1b,e) compared to the TfRMAb-TNFR-treated mice. The hippocampal 6E10-positive puncta number (*p* < 0.05; Figure 1d,e) was significantly higher in the etanercept-treated mice compared to the TfRMAb-TNFR-treated mice and a similar trend was observed for the hippocampal 6E10-positive area (*p* = 0.054; Figure 1c,e).

**Figure 2:**
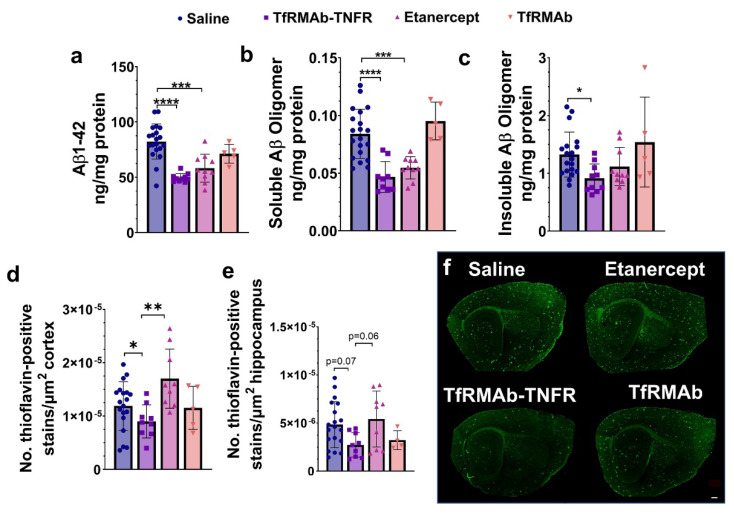
Effect of TfRMAb-TNFR on insoluble Aβ (1-42) and high-molecular-weight Aβ assemblies. TfRMAb-TNFR and etanercept reduced insoluble (Gu-HCl-soluble) Aβ (1-42) (**a**) and soluble (TBS-soluble) high-molecular-weight oligomeric Aβ (**b**) in cerebrum homogenates compared to the saline-treated mice. TfRMAb-TNFR, but not etanercept, significantly reduced insoluble (Gu-HCl-soluble) oligomeric Aβ in the cerebrum homogenates (**c**). TfRMAb-TNFR-treated mice also had a lower number of Thio-S-positive Aβ plaques compared to the saline-treated mice (**d**), and a similar trend was observed in the hippocampus (**e**). Representative sagittal brain section images of Thio-S-stained mature Aβ plaques (**f**). Scale bar = 200 µm. Data are presented as mean ± SD of *n* = 4–20 per treatment group, and data were analyzed using the one-way ANOVA with the Holm Sidak’s post hoc test. * *p* < 0.05, ** *p* < 0.01, *** *p* < 0.001, **** *p* < 0.0001 for the indicated comparisons.

With respect to the cerebrum Aβ ELISAs, both TfRMAb-TNFR and etanercept reduced insoluble (Gu-HCl-soluble) Aβ (1-42) (*p* < 0.001; Figure 2a) and soluble (TBS-soluble) high-molecular-weight Aβ oligomers (*p* < 0.001; Figure 2b) compared with the saline-treated mice. However, only TfRMAb-TNFR significantly reduced the insoluble (Gu-HCl-soluble) high-molecular-weight Aβ species (*p* < 0.05; Figure 2c) and the cortical Thio-S-positive mature Aβ plaque numbers (*p* < 0.05; Figure 2d,f). A similar trend of reduced hippocampal Thio-S-positive mature Aβ plaque numbers (*p* = 0.07; Figure 2e,f) was observed for the TfRMAb-TNFR-treated mice. Like the 6E10-positive Aβ load, etanercept-treated mice had a significantly higher cortical Thio-S-positive mature Aβ plaque number compared with the TfRMAb-TNFR-treated mice (*p* < 0.01; Figure 2d,f) and a similar trend was observed in the hippocampus (*p* = 0.06; Figure 2e,f). TfRMAb alone did not affect Aβ (1-42) or oligomeric Aβ.

### 3.2. Chronic TfRMAb-TNFR Improved Spatial Reference Memory in Aged APP/PS1 Mice

**Figure 3:**
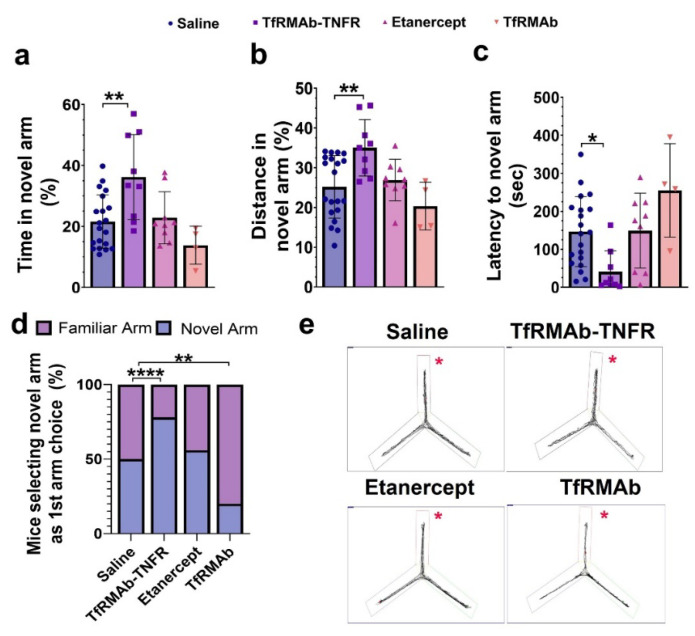
Effect of chronic TfRMAb-TNFR dosing on spatial reference memory using the Y-maze test. TfRMAb-TNFR treatment increased the time spent (**a**) and distance traveled (**b**) in the novel arm and reduced the latency to the novel arm (**c**) compared to the saline-treated mice. More TfRMAb-TNFR-treated mice selected the novel arm as their first choice compared to the saline-treated mice (**d**). Representative trajectory maps of mice in the Y-maze (**e**). The novel arm is shown by the red asterisk. Data are presented as mean ± SD of *n* = 4–20 per treatment group, numerical data were analyzed using the one-way ANOVA with the Holm Sidak’s post hoc test, and nominal data were analyzed using the Fisher’s exact test. * *p* < 0.05, ** *p* < 0.01, **** *p* < 0.0001 for the indicated comparisons.

**Figure 4:**
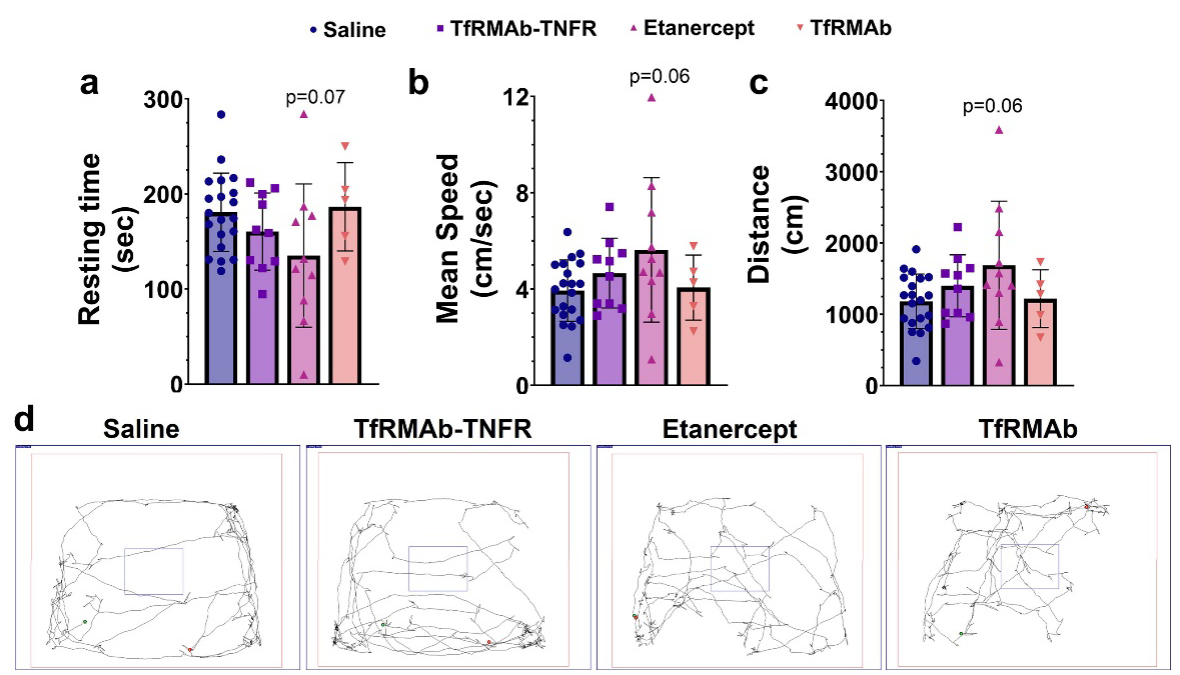
Effect of chronic TfRMAb-TNFR dosing on locomotion and exploration. The resting time (**a**), mean speed (**b**), and distance traveled (**c**) did not differ between any experimental group. The representative trajectory maps show mouse movement in the different groups (**d**). Data are presented as mean ± SD of *n* = 5–20 per treatment group, and data were analyzed using the one-way ANOVA with the Holm Sidak’s post hoc test.

TfRMAb-TNFR-treated mice spent more time (*p* < 0.01; Figure 3a,e) and traveled more distance (*p* < 0.01; Figure 3b,e) in the novel arm compared to the saline-treated mice. TfRMAb-TNFR-treated mice had a reduced latency to enter the novel arm (*p* < 0.05, Figure 3c), and the percentage of mice selecting the novel arm as their first arm choice was significantly higher for the TfRMAb-TNFR-treated mice compared to the saline-treated mice (*p* < 0.0001, Figure 3d). The percentage of mice selecting the novel arm was significantly lower for the TfRMAb-treated mice than for the saline-treated mice (*p* < 0.01, Figure 3d). However, due to the small sample size of the TfRMAb group, these results should be interpreted with caution. To rule out the impact of locomotion on the Y-maze outcome, we performed the open-field test to assess the exploration and locomotion of the APP/PS1 mice. As shown in Figure 4a–d, all three locomotion parameters: resting time (Figure 4a), mean speed (Figure 4b), and distance traveled (Figure 4c), did not differ significantly between any experimental group.

### 3.3. Chronic TfRMAb-TNFR Dosing Did Not Alter APP and BACE-1 Levels but Reduced Microgliosis and Increased Plaque-Associated Phagocytic Microglia

**Figure 5:**
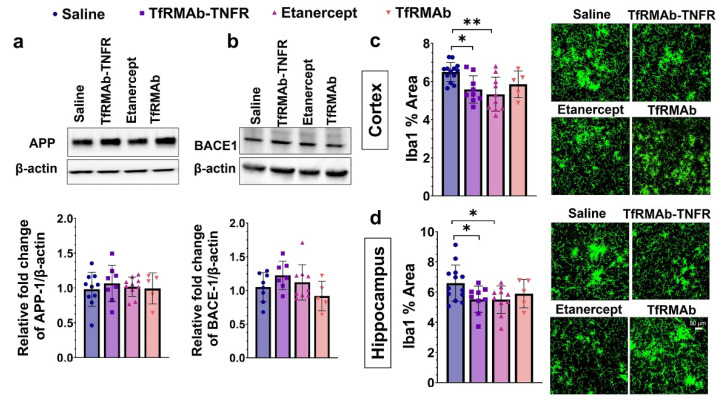
Effect of TfRMAb-TNFR on amyloid precursor protein (APP) and the β-site APP cleaving enzyme (BACE-1) levels, and microgliosis. There was no change in the protein levels of APP (**a**) or BACE-1 (**b**) between experimental groups by Western blotting of the cerebrum homogenates. TfRMAb-TNFR and etanercept reduced cortical (**c**) and hippocampal (**d**) Iba1-positive area. TfRMAb-treated mice showed no change in the Iba1-positive area. Scale bar = 50 µm. Data are presented as mean ± SD of *n* = 5–13 per treatment group, and data were analyzed using the one-way ANOVA with the Holm Sidak’s post hoc test. * *p* < 0.05, ** *p* < 0.01 for the indicated comparisons.

The protein levels of APP and BACE-1 were determined in the cerebrum homogenates and are shown in Figure 5a,b. No differences in APP (Figure 5a) and BACE-1 (Figure 5b) protein levels were found between the experimental groups. Chronic TfRMAb-TNFR or etanercept treatment reduced the Iba1-positive area (microgliosis) in the cortex (*p* < 0.05 for TfRMAb-TNFR and *p* < 0.01 for etanercept; Figure 5c) and the hippocampus (*p* < 0.05 for TfRMAb-TNFR and etanercept; Figure 5d). Chronic TfRMAb dosing did not alter microgliosis in the aged APP/PS1 mice.

**Figure 6:**
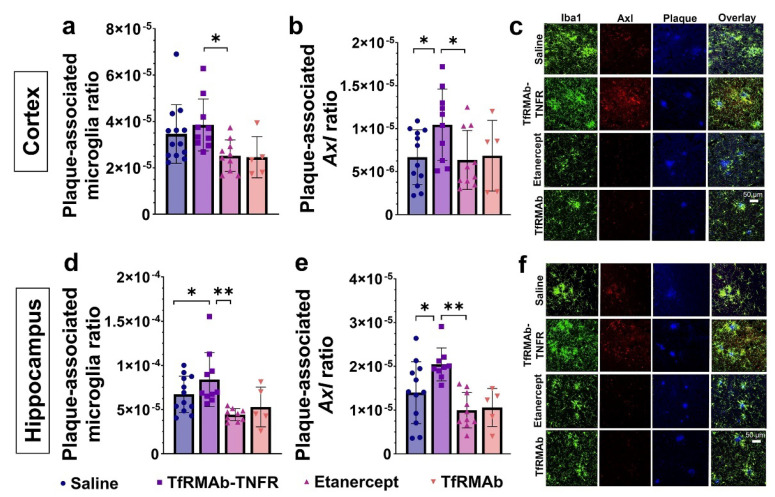
Effect of TfRMAb-TNFR on plaque-associated phagocytic microglia. Cortical plaque-associated microglia (**a**) and plaque-associated Axl-positive phagocytic microglia (**b**) were significantly higher in the TfRMAb-TNFR-treated mice compared with the etanercept-treated mice. Similarly, hippocampal plaque-associated microglia (**d**) and plaque-associated Axl-positive phagocytic microglia (**e**) were significantly higher in the TfRMAb-TNFR-treated mice than in the saline- and etanercept-treated mice. Representative 40× confocal images showing Iba1 (green), Axl (red), and autofluorescent mature Aβ plaques (blue) in the cortex (**c**) and hippocampus (**f**). Scale bar = 50 µm. Data are presented as mean ± SD of *n* = 5–13 per treatment group, and data were analyzed using the one-way ANOVA with the Holm Sidak’s post hoc test. * *p* < 0.05, ** *p* < 0.01 for the indicated comparisons.

To study the association between mature Aβ plaques and microglia, we measured the plaque-associated microglia ratio, which measures the area occupied by the microglia associated with the plaque normalized to the Aβ plaque area. As shown in Figure 6, the plaque-associated microglia ratio was significantly higher in the cortex (*p* < 0.05; Figure 6a,c) and hippocampus (*p* < 0.01; Figure 6d,f) of the TfRMAb-TNFR-treated mice compared to the etanercept-treated mice. The hippocampal plaque-associated microglia were also significantly higher in the TfRMAb-TNFR-treated mice compared to the saline-treated mice (*p* < 0.05; Figure 6d). Interestingly, the plaque-associated Axl ratio, which represents the immunoreactivity of Axl, a phagocytic marker [23], in the plaque-associated microglia, was significantly higher in the cortex (*p* < 0.05 compared to saline and etanercept; Figure 6b,c) and the hippocampus (*p* < 0.05 compared to saline and *p* < 0.01 compared to etanercept; Figure 6e,f) of the TfRMAb-TNFR-treated mice compared to the saline- and etanercept-treated mice. The measured autofluorescent Aβ plaque area was not negatively correlated with the plaque-associated microglia ratio or the plaque-associated Axl ratio suggesting that the higher plaque-associated microglia or Axl ratio in the TfRMAb-TNFR-treated mice and the lower plaque-associated microglia or Axl ratio in the etanercept-treated mice are not driven by the lower and higher autofluorescent Aβ plaque area in the TfRMAb-TNFR and etanercept groups, respectively (Supplementary Materials Figure S1).

### 3.4. TfRMAb-TNFR Treatment Increased BBB Tight-Junction Proteins and Reduced Neuroinflammation

**Figure 7:**
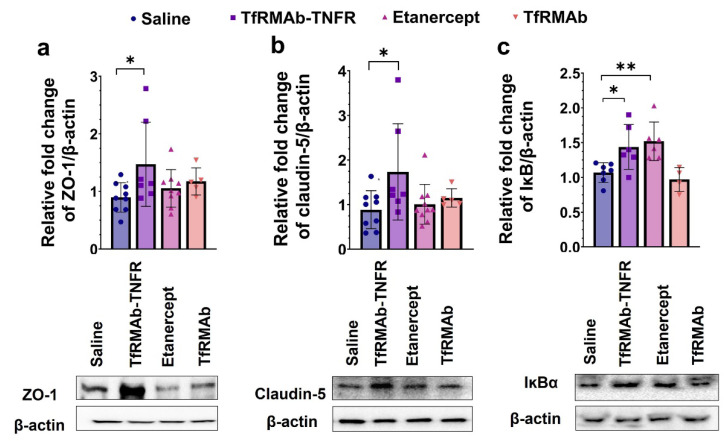
Effect of TfRMAb-TNFR on BBB tight-junction proteins and brain inflammation. Chronic TfRMAb-TNFR dosing in aged APP/PS1 mice significantly increased ZO-1 (**a**) and claudin-5 (**b**) compared to the saline-treated mice measured using Western blotting of the cerebrum homogenates. Both TfRMAb-TNFR and etanercept increased IĸBα in the cerebrum homogenates compared to the saline-treated mice (**c**). Data are presented as mean ± SD of *n* = 4–9 per treatment group, and data were analyzed using the one-way ANOVA with the Holm Sidak’s post hoc test. * *p* < 0.05 and ** *p* < 0.01 for the indicated comparisons.

Chronic TfRMAb-TNFR dosing significantly increased the BBB tight-junction proteins, ZO-1 (*p* < 0.05; Figure 7a) and claudin-5 (*p* < 0.05; Figure 7b), in the cerebrum homogenates of the aged APP/PS1 mice compared with the saline-treated mice. Etanercept or TfRMAb did not alter BBB tight-junction protein expression levels in the aged APP/PS1 mice. Both TfRMAb-TNFR (*p* < 0.05; Figure 7c) and etanercept (*p* < 0.01; Figure 7c) increased IĸBα, a marker of attenuation of TNF-α signaling, in the cerebrum homogenates of the aged APP/PS1 mice. TfRMAb did not alter brain IĸBα protein expression in the aged APP/PS1 mice.

### 3.5. Chronic TfRMAb but Not TfRMAb-TNFR Dosing Altered Hematologic Indices in Aged APP/PS1 Mice

**Figure 8:**
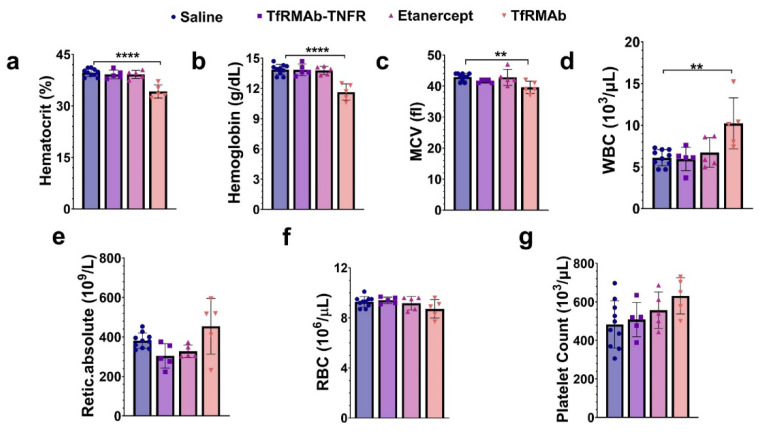
Effect of chronic TfRMAb-TNFR dosing on hematologic parameters. Long-term (ten-week) treatment with TfRMAb alone, but not TfRMAb-TNFR, decreased hematocrit (**a**), hemoglobin (**b**), and mean corpuscular volume (MCV; (**c**)), and elevated white blood cell (WBC; (**d**)) count compared to the saline-treated mice. No change in reticulocytes (**e**), red blood cells (RBC) (**f**), and platelets (**g**) was observed. Etanercept did not alter any hematology parameter. Data are presented as mean ± SD of *n* = 5–10 per treatment group, and data were analyzed using the one-way ANOVA with the Holm Sidak’s post hoc test. ** *p* < 0.01 and **** *p* < 0.0001 for the indicated comparisons.

TfRMAb dosing of 10.7-month-old APP/PS1 mice for ten weeks reduced hematocrit (*p* < 0.0001; Figure 8a), hemoglobin (*p* < 0.0001; Figure 8b), and mean corpuscular volume (MCV, *p* < 0.01; Figure 8c), and increased white blood cell count (WBC, *p* < 0.01; Figure 8d) compared to the saline-treated mice. No change in reticulocytes (Figure 8e), red blood cells (RBC) (Figure 8f), and platelets (Figure 8g) was observed between any treatment group compared to the saline-treated mice. TfRMAb-TNFR or etanercept treatment did not alter any hematology indices following a ten-week treatment compared to the saline-treated mice.

### 3.6. Chronic TfRMAb but Not TfRMAb-TNFR Dosing Increased Splenic Iron Transporters and Iron Levels

**Figure 9:**
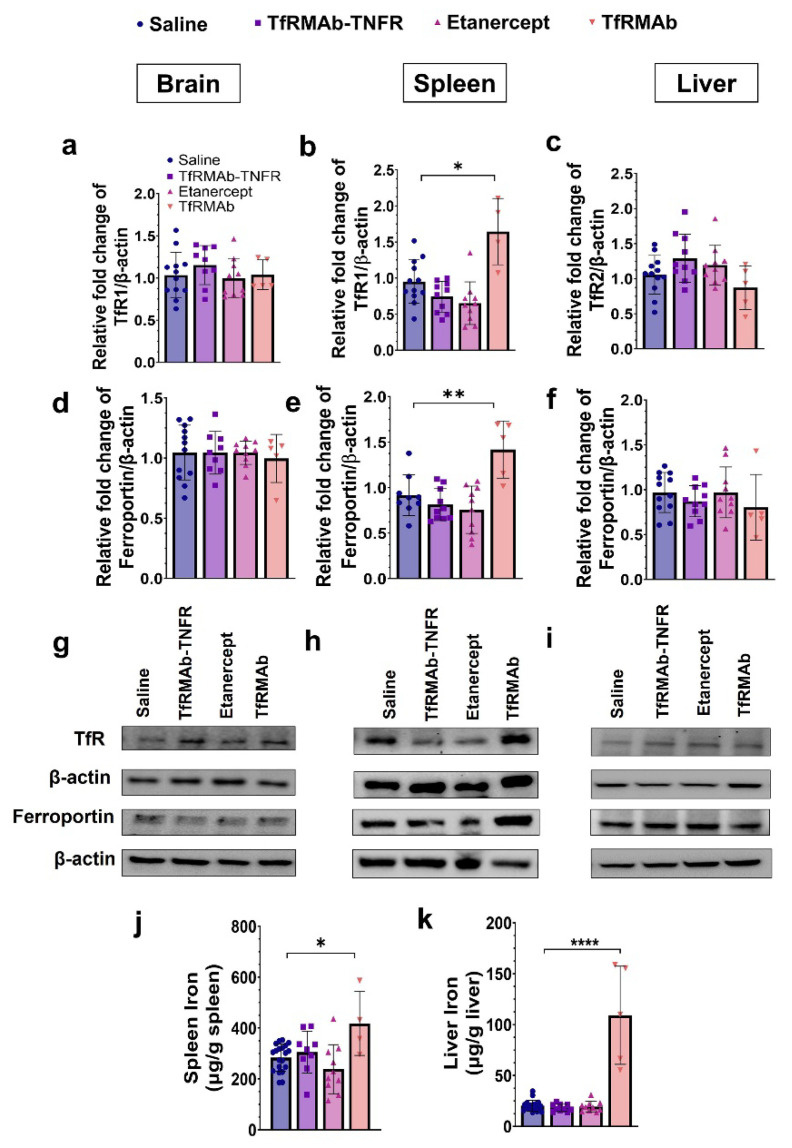
Effect of chronic TfRMAb-TNFR dosing on the expression of iron transporters and tissue iron levels. The protein levels of TfR (TfR1 in the brain and spleen, and TfR2 in the liver) in the brain (**a**), spleen (**b**), and liver (**c**). No significant difference in the brain and liver TfR expression between experimental groups. However, TfRMAb treatment significantly elevated splenic TfR levels compared to the saline-treated mice (**b**). The protein levels of ferroportin in the brain (**d**), spleen (**e**), and liver (**f**). No significant difference in the brain and liver ferroportin expression between experimental groups. However, TfRMAb treatment significantly elevated splenic ferroportin levels compared to the saline-treated mice (**e**). Representative western blot images showing TfR and ferroportin expression in the brain (**g**), spleen (**h**) and liver (**i**). Chronic TfRMAb dosing significantly increased spleen (**j**) and liver (**k**) iron levels. Data are presented as mean ± SD of *n* = 4–20 per treatment group, and data were analyzed using the one-way ANOVA with the Holm Sidak’s post hoc test. * *p* < 0.05, ** *p* < 0.01, and **** *p* < 0.0001 for the indicated comparisons.

The protein levels of TfR (TfR1 in the brain and spleen, and TfR2 in the liver), the principal iron importer, and ferroportin, the major iron exporter, in the brain, spleen, and liver are shown in Figure 9. Brain levels of TfR1 (Figure 9a,g) and ferroportin (Figure 9d,g) did not differ between experimental groups. Similarly, the liver expression of TfR2 (Figure 9c,i) and ferroportin (Figure 9f,i) did not differ between experimental groups. However, chronic TfRMAb dosing significantly increased splenic TfR1 (*p* < 0.05; Figure 9b,h) and ferroportin (*p* < 0.01; Figure 9e,h) levels compared to the saline-treated mice. Chronic TfRMAb dosing also increased splenic (*p* < 0.05; Figure 9j) and liver (*p* < 0.0001; Figure 9k) iron in the aged APP/PS1 mice. TfRMAb-TNFR or etanercept dosing did not alter splenic or liver iron in the aged APP/PS1 mice.

## 4. Discussion

TfRMAb-TNFR is a brain-penetrant biologic TNF-α inhibitor that can sequester both peripheral and brain TNF-α to attenuate or prevent downstream TNF-α signaling [14]. The role of TNF-α in AD pathogenesis has been well studied [6], and our previous work highlighted the protective effects of the brain-penetrant TfRMAb-TNFR in 6-month-old APP/PS1 male mice compared to etanercept, a biologic TNF-α inhibitor with limited brain uptake [17,26]. In the aforementioned proof-of-concept study, we showed that TfRMAb-TNFR reduced Aβ load, which is regarded as the primary initiator of AD [27]. Notably, Aβ deposition in the AD brain is a function of time and progresses with age [27], therefore, an ideal therapeutic for AD may be one that reduces Aβ pathology at both early and late stages of the disease when the Aβ load is full-blown. The advantages of developing such a therapeutic are twofold: first, it will provide an Aβ lowering agent that works at early and late stages of the disease, and second, such investigations will elucidate if Aβ lowering effects at late stages of the disease or following delayed treatment are coincident with cognitive benefits. Therefore, in pursuit of such a therapeutic, in the current study, we initiated TfRMAb-TNFR treatment in older 10.7-month-old APP/PS1 mice, as opposed to treatment initiation in younger 6-month-old mice [17], and continued the treatment till the mice were 13 months old, a stage at which we expect the Aβ pathology to plateau in this mouse model to mimic late stages of AD [28]. Despite the advanced age of the APP/PS1 mice, delayed treatment with TfRMAb-TNFR resulted in an impressive ~50–56% reduction in the 6E10-positive total Aβ load, which is consistent with the reduction seen in the younger 6-month-old APP/PS1 mice [17]. However, chronic equimolar dosing of etanercept did not reduce the 6E10-positive Aβ load, which is consistent with our previous work in the APP/PS1 mice [28] and work of others using the triple transgenic (3xTg) mice showing that peripheral TNF-α inhibitors do not reduce 6E10-positive Aβ load [29].

Amyloidogenic Aβ is formed through the sequential cleavage of the transmembrane APP by β- and γ-secretases resulting in the formation of Aβ monomers [30]. The primary Aβ isoforms associated with AD include Aβ (1-40) and Aβ (1-42) [31], of which, Aβ (1-40) is more abundant while Aβ (1-42) is more prone to aggregation and induces toxicity [32,33]. Monomeric amyloidogenic Aβ isoforms are hydrophobic and self-assemble to form high-molecular-weight oligomers and β-sheet-rich fibrils that form the core of Aβ plaques [34]. The original amyloid cascade hypothesis puts Aβ plaques at the center of AD-neurotoxicity and cognitive decline [35]; however, over the last two decades, there has been a paradigm shift towards the central role of Aβ oligomers in AD pathogenesis and cognitive decline [36]. Therefore, to understand if the Aβ-lowering effects of TfRMAb-TNFR were directed towards a specific Aβ specie, and since the 6E10 antibody detects all Aβ isoforms [37], we measured the levels of different Aβ species: Aβ (1-42), high-molecular-weight Aβ oligomers, and Thio-S-positive β-sheet rich Aβ plaques. Both TfRMAb-TNFR and etanercept reduced insoluble Aβ (1-42) and soluble high-molecular-weight Aβ oligomers. TfRMAb-TNFR treatment significantly reduced insoluble high-molecular-weight Aβ oligomers, and a similar trend was seen with etanercept, but these values did not reach statistical significance. Similarly, TfRMAb-TNFR, but not etanercept, significantly reduced the Thio-S-positive mature Aβ plaques compared to the saline-treated mice and the Thio-S-positive Aβ plaques were lower by almost 50% in the TfRMAb-TNFR-treated mice compared with the etanercept-treated mice. These data collectively show that TfRMAb-TNFR, but not etanercept, results in a significant reduction in total Aβ peptide and mature Aβ plaque load in the aged APP/PS1 mice.

While Aβ accumulation in the brain represents one of the major pathological hallmarks of AD, cognitive impairment represents the main clinical feature of AD [1]. In the APP/PS1 used in the current study, cognitive function is an inverse correlate of Aβ accumulation [38]. Accordingly, TfRMAb-TNFR, which reduced the total Aβ peptide load and Aβ plaque load by half compared to the etanercept treated mice, resulted in a significant improvement in spatial reference memory, implying that the cognitive improvement in the aged APP/PS1 mice may be driven by the lowering of total Aβ peptide and/or Aβ plaque load with TfRMAb-TNFR. Further, increased Aβ load in the brain can be driven by BBB impairment and breakdown, which is an early biomarker of cognitive dysfunction in AD [39,40]. In the current study, apart from reducing Aβ load, TfRMAb-TNFR significantly increased the expression of BBB tight-junction proteins, claudin-5 and ZO-1, while etanercept treatment did not. This increase in BBB tight-junction protein expression concomitant with a reduction in Aβ peptide and/or Aβ plaque load in the TfRMAb-TNFR-treated mice may underlie the improvement in memory of the TfRMAb-TNFR-treated but not of etanercept-treated aged APP/PS1 mice.

Excessive extracellular Aβ deposition in the AD brain is hypothesized to result from an imbalance between Aβ production and Aβ clearance, and TNF-α is implicated in both these processes. Studies show that genetic deletion of TNF-α in 5xFAD mice or its receptor in the APP23 mice reduced Aβ plaque by lowering the levels and activity of the APP cleaving enzymes [41,42]. Further, TNF-α increased the expression of BACE-1 and enhanced APP processing in astrocytes in vitro [43]. In the current study, TfRMAb-TNFR treatment did not alter APP or BACE-1 levels in the cerebrum homogenates, suggesting that the Aβ-lowering effects of TfRMAb-TNFR are not mediated by attenuation of APP processing or Aβ production. However, it must be noted that we did not measure the activity of BACE-1 or the levels of BACE-1 cleavage products in the current study. Given that blockade of TNF-α signaling can reduce BACE-1 activity [41,42], the contribution of lowered BACE-1 activity to Aβ reduction by TfRMAb-TNFR cannot be ruled out.

Microglia are the innate immune cells of the brain, and their primary function is to survey the CNS and respond to pathological stimuli, including Aβ deposits [44,45]. In the AD brain, microglia are found in close proximity to Aβ plaques, become activated in response to the Aβ plaques, and secrete pro-inflammatory cytokines, including TNF-α [44,45]. The secreted pro-inflammatory cytokines can stimulate a self-perpetuating cycle of microglial activation and cytokine release resulting in sustained neuroinflammation [44,45]. In the current study, microgliosis was assessed by Iba1 immunostaining, and chronic treatment with TfRMAb-TNFR reduced the Iba1-positive area in the brains of the aged APP/PS1 mice. A similar reduction in microgliosis was observed in the etanercept-treated mice suggesting that peripheral TNF-α can modulate microglial activation in the CNS. This is consistent with our previous work in the PS19 mouse model of tauopathy and other studies reporting a reduction in microglial activation with attenuation or blockage of peripheral TNF-α signaling [18,41,42] and an increase in microglial activation with increased peripheral TNF-α signaling [46] in AD mouse models.

Besides mounting an inflammatory response to Aβ deposits, microglia surrounding the Aβ plaques can form a protective barrier to reduce neurite dystrophy and perform phagocytic clearance of Aβ plaques [45]. However, despite significant microglial recruitment, there is increased Aβ accumulation in the AD brain, suggesting that microglia cannot effectively clear Aβ deposits in AD [45]. One mechanism resulting in reduced phagocytic activity of microglia is excessive secretion of Aβ-induced pro-inflammatory cytokines [47]. To determine the role of microglia in TfRMAb-TNFR mediated Aβ lowering, we first studied the association of microglia with Aβ plaques in the brains of the aged APP/PS1 mice. Interestingly, despite a reduction in microgliosis, more microglia were associated with Aβ plaques in the TfRMAb-TNFR-treated mice compared to the saline- and etanercept-treated mice. The plaque-associated microglia in the TfRMAb-TNFR-treated mice also had a higher expression of Axl, a phagocytic receptor involved in Aβ plaque phagocytosis [24], compared to the saline- and etanercept-treated mice. These results are consistent with previous findings showing that cytokine suppression can increase microglia-mediated Aβ plaque phagocytosis [47] and that increased expression of Axl on microglial cells is associated with increased Aβ plaque clearance [24]. Therefore, the differential effects of TfRMAb-TNFR and etanercept on lowering Aβ load may be attributed to enhanced plaque-associated phagocytic microglia in the TfRMAb-TNFR-treated mice.

The TfRMAb used in the current study is a high-affinity bivalent antibody [16], and our previous work revealed some alterations in hematology indices and splenic iron with TfRMAb dosing in mice [19]. A single (acute) dose of TfRMAb in mice resulted in severe reticulocyte (immature RBCs) suppression that was not observed with chronic TfRMAb dosing [19]. Reticulocyte suppression with TfRMAbs has been reported for different TfRMAb variants and is suggested to be an immunologic effector function response and related to TfR expression on reticulocytes [19,48]. However, this reticulocyte suppression is short-lived and was not observed with long-term TfRMAb dosing [19], and this was confirmed in the current study, wherein ten-week chronic dosing of TfRMAb was not associated with reticulocyte suppression. However, we did observe a modest but significant decline in other RBC parameters, including hematocrit, hemoglobin, and MCV, following ten weeks of TfRMAb dosing. This contrasts with our results obtained during the 4-week TfRMAb dosing study [19]. RBCs have a life-span of about 40–50 days in a mouse [49,50], and therefore the acute suppression of reticulocytes seen with a single TfRMAb injection will translate into reduced RBC indices around 6–7 weeks after treatment initiation, and a reduction in RBC indices is not likely to appear within four weeks of TfRMAb dosing. This may explain why no changes in RBC indices were seen in the previous four-week TfRMAb dosing study but appeared in the current ten-week dosing study. Longer studies will be needed to determine if this modest reduction in RBC indices with TfRMAb dosing are normalized. Interestingly, these alterations in the hematology indices were TfRMAb specific, and chronic TfRMAb-TNFR dosing did not alter any hematology parameter. This suggests that the fusion of the therapeutic partner alters the safety profile of the TfRMAb, and is consistent with our previous work showing no changes in hematology parameters with chronic eight-week TfRMAb-erythropoietin (EPO) dosing [51]. Similar findings were reported for a humanized TfRMAb chronically dosed in cynomolgus monkeys, wherein humanized TfRMAb-induced effector function was eliminated in monkeys dosed with the humanized TfRMAb fused to a therapeutic enzyme [52]. Stearic hindrance due to the fusion of the therapeutic moiety to the TfRMAb was suggested to interfere with the access of the TfRMAb to the complement proteins that trigger effector function [52]. Chronic etanercept dosing also did not impact the hematology profile of aged APP/PS1 mice.

The TfRMAb is directed towards the mouse TfR1 [16], the principal iron import receptor highly enriched at the BBB and spleen [16,53]. The levels of TfR1 in the liver are low, and the primary hepatic TfR isoform is TfR2 and was measured in the current study [53]. Prior work showed no change in the brain TfR1 expression with chronic four-week TfRMAb dosing in mice [19], and the results from the current investigation are consistent with this. Chronic ten-week TfRMAb or TfRMAb-TNFR dosing did not alter brain TfR1 levels. Additionally, the brain levels of the iron exporter, ferroportin, remained unaltered with chronic TfRMAb or TfRMAb-TNFR dosing. Chronic ten-week TfRMAb dosing, however, elevated TfR1 expression in the spleen of aged APP/PS1 mice, consistent with an increase in splenic TfR1 in young wild-type C57 mice following four-week dosing [19]. This increase in splenic TfR1 correlated with an elevation in the protein level of ferroportin and iron level in the spleen, indicative of altered iron homeostasis in the spleen with chronic TfRMAb dosing. We also studied the impact of chronic TfRMAb dosing on liver TfR2 and ferroportin and did not observe an increase in hepatic TfR2 or ferroportin levels. Interestingly, no correlation between hepatic TfR2 and iron levels was observed, and despite no change in hepatic TfR2 levels, we saw a significant increase in hepatic iron levels with chronic TfRMAb dosing. The reason for this discrepancy is unclear but may be explained by the limited role of hepatic TfR2 in iron uptake [53]. Interestingly, chronic TfRMAb-TNFR dosing did not dysregulate iron transporters or tissue iron levels post ten-week dosing. These findings are intriguing because both TfRMAb and TfRMAb-TNFR are directed towards TfR1, which is highly enriched in the spleen [53]. Accordingly, biodistribution studies following a single injection show enhanced accumulation of TfRMAb-targeted therapeutics, including TfRMAb-TNFR [15], in the spleen [54,55,56]. However, despite the known increased splenic-accumulation of both TfRMAb and TfRMAb-TNFR, only the TfRMAb resulted in iron dysregulation in our hands. Therefore, the effects of TfRMAb on splenic iron and transporters do not appear to be driven by its biodistribution in vivo. These findings highlight the positive impact of the fusion partner in normalizing the TfRMAb-associated altered iron homeostasis, and the mechanism underlying these effects of TfRMAb needs to be further investigated. The positive effect of fusing TfRMAb with a therapeutic partner has been previously observed. While chronic TfRMAb dosing increased its plasma clearance, chronic TfRMAb-TNFR dosing was not associated with altered plasma pharmacokinetics [18,19]. Similarly, TfRMAb-induced effector function was eliminated in the presence of a therapeutic fusion partner, as discussed above [52].

The study has some limitations. First, only aged male APP/PS1 mice were used in the current investigation to compare with prior results obtained from younger 6-month-old male APP/PS1 mice [17]. Therefore, sex differences in the therapeutic effects of TfRMAb-TNFR in APP/PS1 mice were not studied. Second, the sample size for the TfRMAb group is much smaller than the remaining experimental groups. The TfRMAb group was powered for safety studies based on our previous work [19] since the main intent of including the TfRMAb group was to determine if the TfRMAb-related adverse events are also seen with TfRMAb-TNFR. Therefore, the effect of TfRMAb on efficacy endpoints needs to be interpreted with caution. Third, the plaque-associated microglial studies were based on the autofluorescence microscopy of Aβ plaques and did not use classical methods for Aβ plaque detection (6E10 immunostaining and Thio-S). Notably, the use of autofluorescence microscopy to detect Aβ plaques in mouse and human tissue is shown to be a reliable and sensitive method for plaque detection [57,58]. This method provides a chemical- and label-free method for Aβ plaque detection that can be easily combined with Iba1 immunostaining and was therefore used in the current study. Finally, the Aβ oligomer ELISA kit used in the study uses the same detector and capture antibody, which enables the quantification of Aβ dimers and larger Aβ assemblies, thus excluding Aβ monomers [36]. However, it is possible that the Aβ oligomers detected in the Gu-HCl fraction represent undissociated Aβ plaques or larger Aβ assemblies, and the possibility of an overlap between Aβ plaque measurements and insoluble Aβ oligomer measurements cannot be ruled out.

## 5. Conclusions

In conclusion, the results from the current study show that the brain-penetrant TNF-α inhibitor, TfRMAb-TNFR, offers greater therapeutic benefit than its non-brain-penetrant analog, etanercept, even in aged APP/PS1 mice. The superior Aβ-lowering effects of TfRMAb-TNFR were associated with an increase in BBB-tight-junction proteins, plaque-associated phagocytic microglia, and significant memory improvement, effects that were not observed with etanercept. Finally, despite hematology and iron dysregulation with chronic TfRMAb dosing, TfRMAb-TNFR resulted in a stable hematology profile and iron transporter and tissue iron levels. With its stable safety and strong therapeutic profile, TfRMAb-TNFR is a potential therapeutic agent for both the early and late stages of AD.

## Data Availability

All relevant data are within the paper and its supporting files.

## Supplementary Materials

The following supporting information can be downloaded at: https://www.mdpi.com/article/10.3390/pharmaceutics14102200/s1, Figure S1: Scatter plots show no significant correlation between autofluorescent Aβ plaque area and the plaque-associated microglia ratio in the cortex (**a**) and hippocampus (**b**) and plaque-associated Axl ratio in the cortex (**c**) and the hippocampus (**d**) for the biologic TNFI treated groups. Pearson correlation r was used for the analysis.
