# Supplementary material for: Efficacy and Safety of a Brain-Penetrant Biologic TNF-α Inhibitor in Aged APP/PS1 Mice
Source: Pharmaceutics. 2022 Oct 16;14(10):2200. doi: 10.3390/pharmaceutics14102200 (PMC9612380; doi:10.3390/pharmaceutics14102200)
Supplement: Supplementary file 1 [file pharmaceutics-14-02200-s001.zip › pharmaceutics-1805578-supplementary.pdf]

Supplemental Information

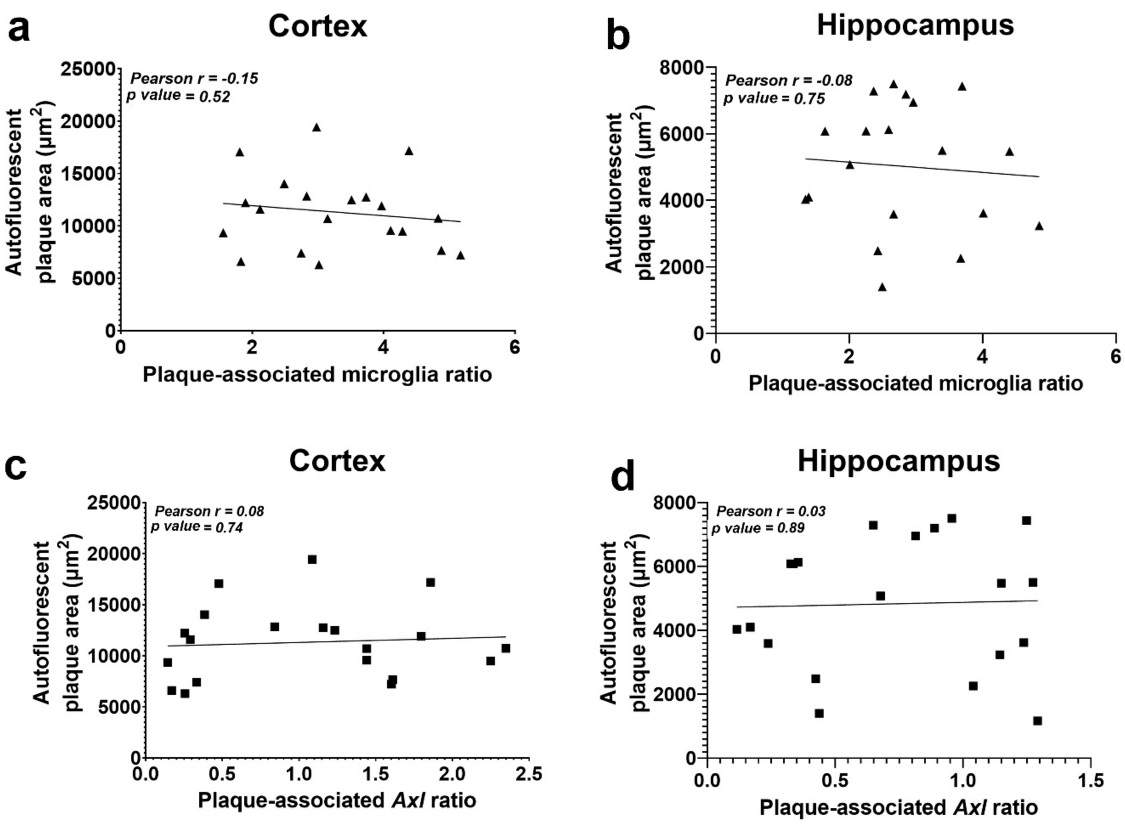

Figure S1: Scatter plots show no significant correlation between autofluorescent Aβ plaque area and the plaque-associated microglia ratio in the cortex (**a**) and hippocampus (**b**) and plaque-associated Axl ratio in the cortex (**c**) and the hippocampus (**d**) for the biologic TNFI treated groups. Pearson correlation r was used for the analysis.
